# HTNV Sensitizes Host Toward TRAIL-Mediated Apoptosis—A Pivotal Anti-hantaviral Role of TRAIL

**DOI:** 10.3389/fimmu.2020.01072

**Published:** 2020-06-19

**Authors:** Qing-Zhou Chen, Xin Wang, Fan Luo, Ning Li, Ni Zhu, Shuang Lu, Yu-xing Zan, Chao-Jie Zhong, Mei-Rong Wang, Hai-Tao Hu, Yong-Zhen Zhang, Hai-Rong Xiong, Wei Hou

**Affiliations:** ^1^State Key Laboratory of Virology, Hubei Province Key Laboratory of Allergy & Immunology, School of Basic Medical Sciences, Institute of Medical Virology, Wuhan University, Wuhan, China; ^2^Department of Microbiology, School of Basic Medical Sciences, Hubei University of Science and Technology, Xianning, China; ^3^Department of Microbiology & Immunology and Sealy Center for Vaccine Development, University of Texas Medical Branch, Galveston, TX, United States; ^4^State Key Laboratory for Infectious Disease Prevention and Control, Collaborative Innovation Center for Diagnosis and Treatment of Infectious Diseases, Chinese Center for Disease Control and Prevention, National Institute for Communicable Disease Control and Prevention, Beijing, China

**Keywords:** HFRS, HTNV, TRAIL, apoptosis, IFN-β

## Abstract

Hantaviruses can cause hemorrhagic fever with renal syndrome (HFRS) in Eurasia and have led to public health threat in China. The pathogenesis of HFRS is complex and involves capillary leakage due to the infection of vascular endothelial cells. Accumulating evidence has demonstrated that hantavirus can induce apoptosis in many cells, but the mechanism remains unclear. Our studies showed that Hantaan virus (HTNV) infection could induce TNF-related apoptosis-inducing ligand (TRAIL) expression in primary human umbilical vein endothelial cells (HUVECs) and sensitize host cells toward TRAIL-mediated apoptosis. Furthermore, TRAIL interference could inhibit apoptosis and enhance the production of HTNV as well as reduce IFN-β production, while exogenous TRAIL treatment showed reverse outcome: enhanced apoptosis and IFN-β production as well as a lower level of viral replication. We also observed that nucleocapsid protein (NP) and glycoprotein (GP) of HTNV could promote the transcriptions of TRAIL and its receptors. Thus, TRAIL was upregulated by HTNV infection and then exhibited significant antiviral activities *in vitro*, and it was further confirmed in the HTNV-infected suckling mice model that TRAIL treatment significantly reduced viral load, alleviated virus-induced tissue lesions, increased apoptotic cells, and decreased the mortality. In conclusion, these results demonstrate that TRAIL-dependent apoptosis and IFN-β production could suppress HTNV replication and TRAIL treatment might be a novel therapeutic target for HTNV infection.

## Introduction

Hantaviruses (family *Hantaviridae*, order *Bunyavirales*) are enveloped, negative-sense viruses and contain a tripartite RNA genome (S, M, and L segments), which encodes the nucleocapsid protein, glycoproteins (G1 and G2), and the RNA-dependent polymerase protein, respectively (1). Hantavirus infection causes two major diseases in infected human individuals: hemorrhagic fever with renal syndrome (HFRS) and hantavirus cardiopulmonary syndrome (HCPS) (2). HFRS is caused by the Old World hantaviruses, including Hantaan virus (HTNV), Seoul virus (SEOV), Dobrava virus (DOBV), and Puumala virus (PUUV) (3), while Sin Nombre hantavirus (SNV), Andes virus (ANDV), New York-1 virus (NY-1V), and other New World hantaviruses presented in the Americas are the prominent cause of HCPS disease (3, 4). More than 90% of HFRS occur in Eurasian area, and China serves especially as the most seriously affected country (5). As a global disease, there are no effective treatments approved by the U.S. Food and Drug Administration for hantavirus infection, and the knowledge on the viral replication and pathogenesis is limited.

Hantaviruses mainly infect the vascular endothelial cells, leading to capillary leakage. Since hantavirus infection is not lytic, the endothelial cell responses are deemed to be the key player in hantavirus pathogenesis (6). Apoptosis is a fundamental biologic process in the maintenance of tissue homeostasis, which has been observed in both Old and New World hantavirus infections. Apoptosis induced by hantaviruses was firstly reported in 1999, with evidence in HTNV- and Prospect Hill virus (PHV)-infected Vero-E6 cells (7). Since then, the presence of apoptosis has been highlighted in different host cells infected by various subtypes of hantaviruses. Studies suggested that apoptosis and cytopathic effects have occurred in HTNV-, SEOV-, and ANDV-infected HEK293 cells (8). Tula virus (TULV) infection can induce apoptosis through activating caspase-8 (9) and triggering pro-apoptotic signals of endoplasmic reticulum (ER) stress (10). The nucleocapsid protein of PUUV can interact with the Fas-mediated apoptosis enhancer Daxx (Fas death domain-associated protein) in 293T cells, which denotes a direct connection between cellular apoptotic factor and a hantaviral component (11). Researchers further revealed that hantavirus infection interferes with DAXX-mediated apoptosis in ANDV- and HTNV-infected HUVECs by activation of the interferon-stimulated nuclear transcription factor pro-myelocytic leukemia protein (PML) (12). Moreover, apoptotic phenomena have also been observed in hantavirus-infected patients, who have shown an increased level of Fas/FasL, activation of the first-step apoptosis associated protein (caspase-2,−8, and−9), and the initiation of the apoptosis effector molecules (caspase-3,−7, and−10) in peripheral blood mononuclear leucocytes (PBML) during acute the and convalescent phases of the hantavirus infection (13). The caspase-cleaved cytokeratin-18 (CK18), an epithelial cell apoptosis marker, has been found to be increased in the serum samples obtained from PUUV-infected HFRS patients (14). However, Hardestam et al. argue that hantaviruses do not induce apoptosis in confluent Vero-E6 and A549 cells and may induce a very low level of apoptosis in dividing cells (15). Ontiveros et al. show that the cellular apoptotic responses are inhibited by exogenous-expressing HTNV N protein in Hela cells (16). Gupta et al. demonstrated that HTNV and ANDV [multiplicity of infection (MOI) = 1] inhibited NK cell-mediated cytotoxic granule-dependent induction of apoptosis in the endothelial co-cultured with NK cells. They also showed that HTNV and ANDV (MOI = 0.01) inhibited staurosporine-induced apoptosis at lower viral titer (17). It is possible that different hantaviruses would induce apoptosis to varying degrees (7–9, 13). Nevertheless, the precise mechanism how hantavirus regulates a specific apoptotic response in endothelial cells *per se* remains obscure.

The tumor necrosis factor (TNF)-related apoptosis-inducing ligand (TRAIL) is a member of TNF superfamily (18) and is able to induce apoptosis in a variety of cancer cells, transformed cells, and virus-infected cells without significantly affecting normal cells (19). TRAIL promotes apoptosis by binding to two death-inducing receptors, including TRAIL-R1 (death receptor 4, DR4), and TRAIL-R2 (death receptor 5, DR5). The other three inhibitory receptors, TRAIL-R3 (decoy receptor 1, DcR1), TRAIL-R4 (decoy receptor 2, DcR2), and TRAIL-R5 (OPG), can competitively bind to TRAIL, resulting in the inhibition of apoptosis signal (19, 20). Although TRAIL-based anti-tumor therapeutic approaches have brought great interest to a clinical application, its possibly crucial role in viral infection has just begun to be elucidated. A variety of viruses, such as cytomegalovirus (CMV) (21), hepatitis B virus (HBV) (22), hepatitis C virus (HCV) (23), respiratory syncytial virus (RSV) (24), reovirus (25), and human immunodeficiency virus (HIV) (26) can upregulate TRAIL expression and sensitize host cell to TRAIL-mediated apoptosis, which provides the host cells an “antiviral state.” In contrast, some other viruses, including adenovirus (Adv) (27), herpes simplex virus (HSV) (28), human papillomavirus (HPV) (29), and Epstein-Barr virus (EBV) (30), may evolve strategies to escape TRAIL-mediated apoptosis, which facilitates viral replication and transmission and enhances viral pathogenesis. Notably, the TRAIL expression can be regulated by cytokines, especially interferons (IFNs), through paracrine and autocrine signaling (31–33). The antiviral response against encephalomyocarditis virus (ECMV) by NK cells depends on TRAIL expression enhanced by IFN-α/β production after viral infection (32). These correlations between high IFN production and elevated levels of cell surface TRAIL or circulating TRAIL are also observed in CMV-infected NK cells and HCV-infected DC/CD8^+^ T cells (20, 32). It is reported that the TRAIL expression is upregulated in the peripheral blood mononuclear cells (PBMCs) from HFRS patients, and the levels of sTRAIL circulating in plasma are also elevated for patients in the acute phase of HFRS (34). However, the elaborate mechanism of how TRAIL regulates hantavirus replication is unclear; at least, there are no related animal experiments on the role of TRAIL in hantavirus infection to date.

In this study, we found that HTNV increased TRAIL expression, activated caspase-8-dependent apoptosis signaling, and induced type I IFN production in primary HUVECs. Knockdown of TRAIL could inhibit apoptosis and enhance the production of an HTNV virion along with the decline of IFN-β production. As anticipated, exogenous TRAIL enhanced apoptosis and inhibited HTNV replication. Thus, TRAIL is inducible by HTNV infection and has significant antiviral activities *in vitro*, which was further confirmed in the HTNV-infected suckling mouse model—that TRAIL treatment significantly reduced viral load, alleviated virus-induced tissue lesions, and decreased mortality. Together, these results demonstrated that TRAIL-dependent apoptosis can suppress HTNV replication and TRAIL may be an important and novel therapeutic target for HTNV infection.

## Materials and Methods

### Cells, Virus, and Reagent

Human umbilical vein endothelial cells (HUVECs) were purchased from ScienCell Research Laboratories (Cat No: 8000) and cultured in Endothelial Cell Medium (Cat No: 1001, ScienCell Research Laboratories), which contains 5% fetal bovine serum (FBS), 1% endothelial cell growth supplement, and 100 U/mL penicillin and 100 μg/mL streptomycin in a humidified incubator with 95% air and 5% CO_2_. HUVECs were grown on gelatin-coated plates and used for no more than 6 passages. Vero-E6 cells were maintained in our laboratory as described before (35) and used for propagation of HTNV.

HTNV 76-118 was obtained from Institute of Virology, Chinese Center for Disease Control and Prevention (CDC). The titers of the virus stock were determined to be 10^5.00^/mL by focus assay after the immunofluorescence staining of hantavirus nucleocapsid protein within cells, as previously described (36). HUVECs were infected at an MOI of 1 in all experiments.

Recombinant human TRAIL (rTRAIL) was obtained from R & D Systems (Cat No: 375-TL). Cells were treated with rTRAIL at a concentration of 40 ng/mL for different days after viral infection.

### Lentivirus-Mediated TRAIL-Specific Short Hairpin RNA (shRNA)

Four lentiviral vector GV248 expressing TRAIL-specific shRNA was purchased from Shanghai Genechem Co., Ltd. The targeted shRNAs against human TNFSF10 gene (Gene Bank Accession NM_003810) were designed as follows: shRNA19778, 5′-AACAAATGAGCACTTGATA-3′; shRNA 19779, 5′-ACAAACAAATGGTCCAATA-3′; shRNA 19780, 5′-ATTT CTACAGTTCAAGAAA-3′; and shRNA 19781, 5′-TGTAACTTACGTGTACTTT-3′. The four shRNA-expressing plasmids were tested for their abilities to silence TRAIL in 293T cells (Figures S1A,B), and we used the most effective of those, shRNA 19781, to establish further lentivirus packaging. The lentiviruses were produced by co-transfecting HEK293T cells at 70–80% confluence with recombinant lentiviral expression plasmid GV248 and packing plasmids (pHelper 1.0 including gag/pol and pHelper 2.0, including VSVG). After 48 h, the supernatants were harvested, concentrated and titered by counting GFP-positive cells after serial dilutions. The final lentiviral particles were termed as TRAIL^KD^-LV or TRAIL^CTL^-LV, with average titers of 8 × 10^8^ TU/mL. The HUVECs were transduced with lentiviral particles at an MOI of 1. The transduction efficiency was observed and recorded under a fluorescence microscope (Nikon TE2000) 48 h post infection (more than 80% transduction efficiency, Figure S1). The interference efficiency was determined by quantitative real-time PCR (qRT-PCR) and Western blot.

### Quantitative Real-Time PCR

Total cellular RNA was extracted from cultured cells with TRIZOL (Invitrogen) according to the experimental requirements. Complementary DNA was synthesized by using random primer and Moloney murine leukemia virus (M-MLV) reverse transcriptase (Promega). Real-time PCR was carried out by using a SYBR green real-time PCR master mix (TOYOBO) under a CFX96™ instrument as follows: 95°C for 3 min, 40 cycles of 95°C for 10 s, 60°C for 10 s, and 72°C for 15 s. The genes expression was measured in this study are listed as follows: HTNV S gene, forward, 5′-TCTAGTTGTATCCCCATCGACTG-3′, reverse, 5′-ACATGCGGAATACAATTATGGC-3′; human TRAIL gene, forward, 5′-GAGCTGAAGCAGATGCAGGAC-3′, reverse, 5′- TGACGGAGTTGCCACTTGACT-3′; human TRAIL-DR4 gene, forward, 5′-TACGCCCTGGAGTGACATCG−3′, reverse, 5′-CCACAACCTGAGCCGATGC-3′; human TRAIL-DR5 gene, forward, 5′-AAGACCCTTGTGCTCGTTGT-3′, reverse, 5′-AGGTGGACACAATCCCTCTG-3′; human TRAIL-DcR1 gene, forward, 5′-CTGCCAGTCCTAGCTTACTCTGC-3′, reverse, 5′-GGGTTACAGGCTCCAGTATGTTCT-3′; human TRAIL-DcR2 gene, forward, 5′-AAGGCATCTGCTCAGGTGGT-3′, reverse, 5′-AAGTATCTGTTACTCAGGGTCTCGTT-3′; human TRAIL-DcR2 gene, forward, 5′-AAGGCATCTGCTCAGGTGGT-3′, reverse, 5′-AAGTATCTGTTACTCAGGGTCTC GTT-3′; human caspase-3 gene, forward, 5′-CATGGAAGCGAATCAATGGACT-3′, reverse, 5′-CTGTACCAGACCGAGATGTCA-3′; human caspase-8 gene, forward, 5′-TTTCTGCCTACAGGGTCATGC-3′, reverse, 5′-CTGTACCAGACCGAGATGTCA-3′; human caspase-9 gene, forward, 5′-CTTCGTTTCTGCGAACTAACAGG-3′, reverse, 5′-GCACCACTGGGGTAAGGTTT-3′; human IFNB1 gene, forward, 5′-CGCCGCATTGACCATCTA-3′, reverse, 5′-GACATTAGCCAGGAGGTTCTCA-3′; human IRF3 gene, forward, 5′-ACCAGCCGTGGACCAAGAG-3′, reverse, 5′-TACCAAGGCCCTGAGGCAC-3′; human IRF7 gene, forward, 5′-CGACATCGAGTGCTTCCTTATG-3′, reverse, 5′-ACTGGGTTCTAGGCGGGC-3′; human GAPDH gene, forward, 5′-GGTGGTCTCCTCTGACTTCAACA-3′, reverse, 5′-GTTGCTGTAGCCAAATTCGTTGT-3′; mouse GAPDH gene, forward, 5′-ACCCAAAGACTGTGGATGG-3′, reverse, 5′-ACACATTGGGGGTAGGAACA-3′. The different gene levels were normalized to that of the internal control gene GAPDH level in each experiment.

### Immunofluorescence Assay

Immunofluorescence assay (IFA) detection of HTNV antigens was performed as described before (36). HUVECs were cultured on coverslips or 96-well plates. After treatment, the cells were fixed with ice-cold paraformaldehyde for 15 min at 4°C, washed with fluorescence PBS (FPBS), and blocked with 5% bovine serum albumin (BSA) in FPBS for 30 min. Cells were then incubated with monoclonal antibody against HTNV/76-118 (ab20309, 1:100, Abcam) for 1.5 h at room temperature or 4°C overnight. Then, the samples were incubated with DyLight 488 AffiniPure Goat Anti-Mouse IgG (H+L) (A23310, 1:100, Abbkine) for 1.5 h at room temperature. Nuclei were stained with Hoechst33258 (1:10,000, Beyotime) for 15 min at room temperature. The images were captured under a Nikon TE2000 fluorescence microscope or a confocal microscope (TCS SP2 AOBS MP, Leica).

### Western Blot Analysis

After the required treatments, protein extracts were prepared as follows. Briefly, cells were washed with cold PBS and lysed in RIPA buffer containing PMSF (Wuhan Goodbio, Lot: 170118). Equal amounts of the protein samples were resolved by 12% SDS-PAGE and transferred to an Immobilon-P membrane (Biosharp). Each membrane was, respectively, probed with a polyclonal antibody against TRAIL protein (Abcam ab2435, 1:1,000), human DR4 (Flarebio CSB-PA002191, 1:1,000), human DR5 (Flarebio CSB-PA08165A0Rb, 1:1,000), human DcR1 (Abcam ab133658, 1:2,000), human DcR2 (Cusabio E0425R1, 1:1,000), human caspase-3 (Beyotime AC033, 1:1,000), human caspase-8 (Beyotime AC056, 1:1,000), human caspase-9 (Beyotime AC062, 1:1,000), human cleaved-PARP (Beyotime AP102, 1:1,000), a mouse monoclonal antibody against GAPDH (Tianjin Sungene Biotech DKM9002T, 1:5,000), and IRF3 (ABclonal Q14653, 1:1,000), IRF7 (ABclonal Q92985, 1:1,000). The blots were incubated with HRP-conjugated secondary antibodies and developed by an ECL detection kit (Wuhan Goodbio Technology Co. LTD). The results were visualized using an Image Station 4000R (Kodak) and the signals were quantified using carestream software. The calculation method of protein grayscale ratio is based on the reference (37).

### Plasmids Construction and Luciferase Activity Assay

HTNV S and M segment expression plasmids were constructed on the basis of pSicoR-flag-cherry, which was reformed from pSicoR-GFP. Reporter plasmids were constructed using the pGL3 vector containing a firefly luciferase open reading frame (Promega). The human DR4 and DR5 promoters were kindly provided by Dr. Deyin Guo from Zhongshan University. The human TRAIL, DcR1 and DcR2 promoters were cloned from Human Genomic DNA (Cwbio CW0565S). PCR for the promoters was performed with following primers: TRAIL (sense: 5′-CGGCTAGCCGACTCTTGTAACTCCTCAAATCAC-3′, antisense: 5′-ATCTCGAGGATCCTGTCAGAGTCTGACTGCT-3′), DcR1 (sense: 5′-CGGCTAGCAACTCTATGACCAAGACGTTGAG-3′, antisense: 5′-ATCTCGAGTAACGGTAGGAAGCGCTCCTTCA-3′), and DcR2 (sense: 5′-CGGCTAGCTCTGGTCTATACTGTGTGGTCC-3′, antisense: 5′-ATCTCGAGCAATCAGAAATCGTCCCCGTAGT-3′). Two days post-transfection, the 293T cells were washed once with cold PBS, and 80 μL of lysis buffer (Promega) was then added to each well of the 24-well plate. Sixty microliter of sample were mixed with 15 μL luciferase assay substrate (Promega). Luciferase activity was typically measured for 10 s using a luminometer (Turner Designs TD-20/20). Assays were performed in triplicate, and the results are expressed as the mean ± standard deviation (SD) of relative luciferase activity.

### TdT-Mediated dUTP Nick-End Labeling (TUNEL) Assay

Apoptosis was measured using the terminal deoxynucleotidyl transferase (TdT)-mediated dUTP nick-end labeling (TUNEL) assay, as previously reported (38). Cell apoptosis was assessed by TUNEL assay using the one-step TUNEL apoptosis assay kit (Beyotime, C1088). HUVECs and tissue sections were washed with PBS and fixed with 4% polyformaldehyde and then incubated with TUNEL reaction mixture (Cat no: 11684817910, Roche Applied Science) according to the instructions. The fixed cells and tissue sections were treated with 0.3% Triton X-100 to increase cell membrane permeability and stained the nucleus with Hoechst. Fluorescent images were captured by confocal fluorescence microscopy (Leica-LCS-SP8-STED, Leica). Eight horizons of each group were selected randomly to calculate cell apoptosis rate. TUNEL apoptotic images were analyzed by Image pro-plus 6.0 software.

### Flow Cytometry

HTNV-infected HUVECs were resuspended in PBS. Then cells were stained with anti-human DR4 -PE (Biolegend, 307205), anti-human DR5 -PE (eBioscience, 12-9980-41), anti-human DcR1 -PE (Biolegend, 307005) and anti-human DcR2 -FITC (Invitrogen, A15752) in the dark for 30 min at 4°C, and anti-human IgG-PE/FITC isotypes were set as control. The cell region was gated-based FSC and SSC characteristics, and positive cells were defined according to the PE or FITC signaling from unstained group (Figure S2). FACS analysis was performed using BD FACSAria III and FlowJo software.

### *In vivo* Experiment

Pregnant BALB/c mice were purchased from the Animal Research Center of Hubei province (Certificate No. SCXK 2015-0018, Hubei) and were fed in the facility of ABSL-3 Laboratory of the Animal Research Center at Wuhan University under specific pathogen-free conditions. Animal welfare and protocols were in compliance with the guidelines of the Institutional Animal Care and Use Committee (Wuhan, China). Pregnant mice were observed daily, and births were timed to the nearest day. The suckling mice (1- to 2-days old) were intracranially (i.c.) inoculated with 10^−1^ to 10^−4^ viruses to determine the LD_50_ (50% lethal dose) of HTNV, which was 10^5.37^/mL.

The suckling mice were infected i.c. with 20 μL viral suspension containing 50 × LD_50_ of HTNV. Any death occurring 24 h post-infection (hpi) was considered traumatic injury and excluded from the following experiment. The mice were randomly divided into the following two groups that were intraperitoneally administered either solvent control (PBS) or rTRAIL (0.5 mg/kg/day) for 7 days. The normal control received no treatment. Brain, lung, and kidney were aseptically dissected from the animals at 3, 6, 9, 12, 14, and 35 days post-infection (dpi) and divided into four parts for the subsequent experiments, including qRT-PCR, Western blot, H & E staining, and TUNEL assay.

### Statistical Analysis

Data were representative of three independent experiments and were expressed as the mean ± SD. Student's *t*-test or one-way analysis of variance (ANOVA) was used to compare the statistical differences between groups. All statistical analyses were performed with SPSS 20.0 (SPSS Inc., USA). The difference was considered statistically significant at *p* < 0.05.

## Results

### HTNV Infection Induces TRAIL-Dependent Extrinsic Apoptosis in HUVECs

**Figure 1:**
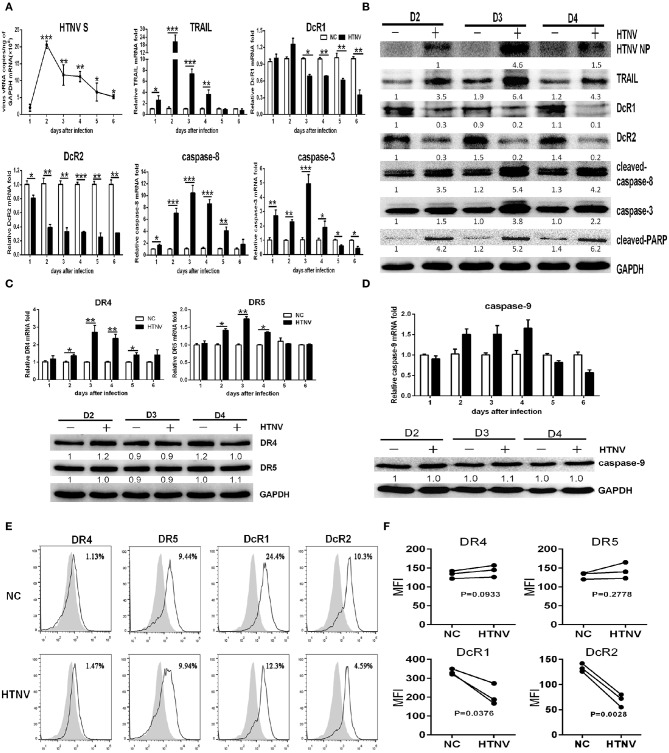
HTNV induces TRAIL-dependent apoptosis in primary HUVECs. HUVECs were infected with/without HTNV 76-118 (MOI = 1) for 2 h and followed by qRT-PCR and Western blot at different days to measure TRAIL-related apoptosis factors. **(A)** HTNV S, TRAIL, DcR1, DcR2, caspase-8, and caspase-3 mRNA in HTNV-infected HUVECs. **(B)** HTNV NP and TRAIL-related apoptosis proteins expression in HTNV-infected HUVECs. **(C)** TRAIL-DR4/DR5 and **(D)** caspase-9 mRNA and protein expression during HTNV infection. The mRNA results shown are the average of three replicates; values represent the mean ± SD (^*^p < 0.05; ^**^p < 0.01; ^***^p < 0.001). Proteins were quantified using carestream software and compared with the normal control group of 2 dpi. Data were from one of three experiments with similar results. The numbers represented the relative density of the bands relative to the corresponding control. **(E)** Histogram showing DR4, DR5, DcR1, and DcR2 surface expression on HTNV-infected and uninfected HUVECs at 2 dpi. White histograms indicate fluorescent labeling with receptor-specific monoclonal antibodies, and gray histograms show background labeling with isotype-matched control antibody in the same group. The experiment was repeated three times and data represent one of three separate experiments. **(F)** Data showing the MFI of DR4, DR5, DcR1, and DcR2 surface expression on HTNV-infected relative to the uninfected HUVECs at 2 dpi. The MFI of TRAIL receptors was assessed by FlowJo software. The experiment was repeated three times. The dots represented the average of each experiment and data were analyzed by a two-tailed, two-sample student *t*-test (*p*-values were indicated).

To evaluate potential role of TRAIL-dependent apoptotic signaling induced by HTNV infection, we first examined dynamic expression of TRAIL and its receptors by qRT-PCR and Western blot. As shown in Figure 1A, after infection by HTNV 76-118 (MOI = 1), the viral RNA level in HUVECs reached peak at 2 dpi and declined thereafter. IFA assay indicated that more than 85% of HUVECs were HTNV infected at 3 dpi (Figure S3). The TRAIL mRNA level in HTNV-infected HUVECs reached peak at 2 dpi (fold change > 20, Figure 1A) and the protein at 3 dpi, which was consistent with those of HTNV vRNA and protein expression (Figures 1A,B). Meanwhile, the transcripts of pro-apoptotic receptors (DR4 and DR5) displayed similar trend with peak at 3 dpi (about 1.5- to 3-fold increase), while their protein level didn't significantly change (Figure 1C). Furthermore, HTNV decreased the mRNA and protein expression of anti-apoptotic receptors (DcR1 and DcR2) (Figures 1A,B). In addition, we also detected changes of TRAIL receptors in HUVECs surface after viral infection by flow cytometry at 2 dpi (Figure 1E). The percentage of positive cells and median fluorescence intensity (MFI) data show that the receptors of DR4 and DR5 in HUVECs surface show slight change after HTNV infection (panels 1 & 2; Figures 1E,F). It is worth noting that the distribution of DcR1 and DcR2 in HUVECs surface after HTNV infection show a downward trend (panels 3 & 4, Figures 1E,F), which is consistent with gene and protein results. These results indicated that HTNV infection may promote TRAIL-dependent apoptotic signaling pathway in primary HUVECs.

The HTNV-induced apoptosis in HUVECs has not been described well yet. We investigated TRAIL-related extrinsic pathway and the mitochondrial intrinsic pathway in HTNV-infected HUVECs. The mRNA expressions of caspase-8 and caspase-3 increased significantly at 1 dpi (Figure 1A), which reached a peak at 3 dpi, after the highest expressions of HTNV vRNA and TRAIL mRNA (Figure 1A). The HTNV infection also induced a cleaved-caspase-8 increase in the form of 43-kDa fragments. As shown in Figure 1B, immunoblot analysis showed that HTNV infection induced the expression of caspase-3 and cleaved-PARP at 3 dpi, among which cleaved-caspase-8 activated caspase-3 and, in turn, cleaved the subsequent substrate of PARP (39). However, the mRNA and protein expression of caspase-9 remained unchanged during HTNV infection (Figure 1D). Our results indicated that HTNV may trigger extrinsic caspase-8-dependent apoptotic pathway in HUVECs, rather than mitochondrial caspase-9-dependent.

### HTNV Structural Proteins Trigger the Expressions of TRAIL and Its Receptors

**Figure 2:**
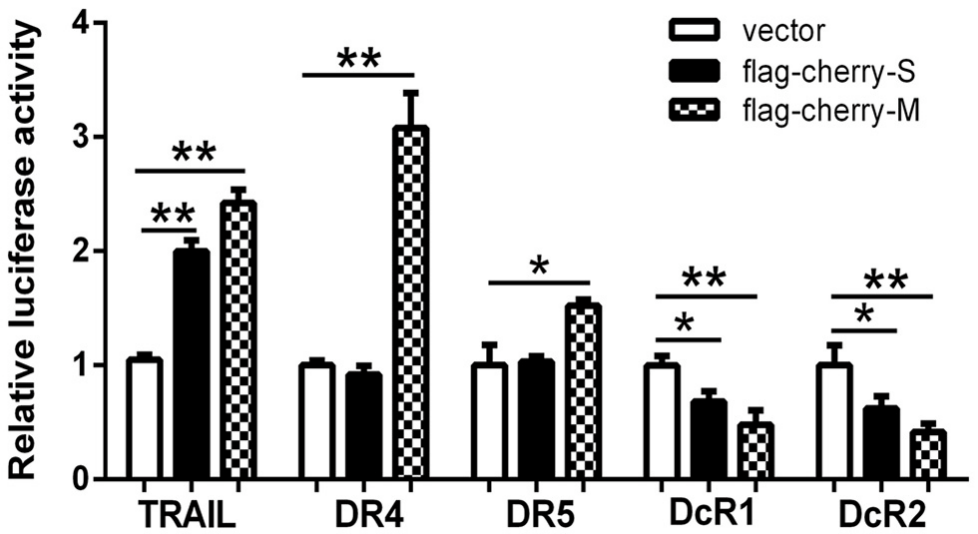
The hantaviral structural proteins affect the transcriptional activity of the promoters of TRAIL/TRAIL-Rs. Firefly luciferase reporter plasmids (400 ng) containing TRAIL, DR4, DR5, DcR1, or DcR2 promoter and hantaviral structural plasmids (400 ng) including pSicoR (vector), pSicoR-S, or pSicoR-M were co-transfected into HEK293T cells to detect the promoter activity. Luciferase activity represented the effect of hantaviral structural proteins on the promoters of TRAIL/TRAIL-Rs. The data were presented with the mean ± SD from three independent experiments of relative luciferase activity (^*^p < 0.05; ^**^p < 0.01).

To elucidate the mechanism of how HTNV increases the expressions of TRAIL and its receptors, we constructed several reporter plasmids containing the promoter regions of these genes. Interestingly, overexpression of the S or M segments of HTNV in 293T cells induced TRAIL expression (Figure 2). Of note, the S segment of HTNV failed to induce the expressions of DR4 and DR5, while the M segment could upregulate their expressions, indicating that the HTNV glycoprotein was responsible for upregulation of DR4 and DR5. Consistently, S or M segment of HTNV could downregulate the transcription of DcR1 (31.96 ± 5.27 and 37.9 ± 6.13% decrease, respectively, *p* < 0.05) and DcR2 (52.2 ± 8.98 and 58.64 ± 4.38% decrease, respectively, *p* < 0.01). The above findings indicate that the expression of individual structural proteins (S or M segment) could enhance the transcriptions of TRAIL and its receptors, and glycoprotein seemed to be more efficient than nucleocapsid protein. Since hantaviruses are known to replicate in the cytoplasm, we hypothesize that HTNV NP or GP may interact with importin or another transport or induce transcription factors expression, which may further affect the promoter activity of TRAIL and its receptor.

### TRAIL-Dependent Apoptosis Suppresses HTNV Replication

**Figure 3:**
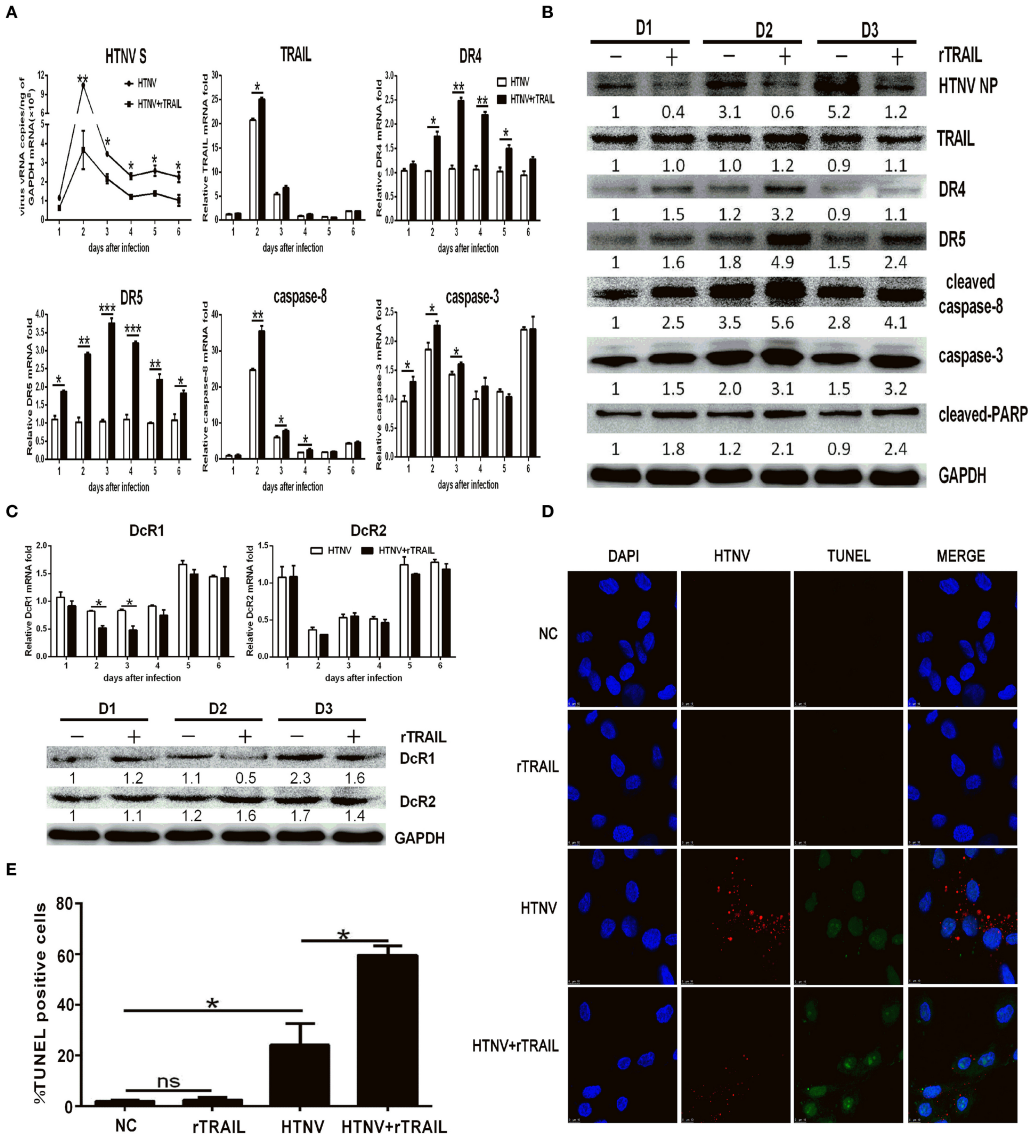
Exogenous rTRAIL inhibits HTNV infection by enhancing caspase-8-dependent apoptosis. HUVECs were infected with HTNV76-118 (MOI = 1) for 2 h, treated with/without rTRAIL (40 ng/mL), and then followed by qRT-PCR and Western blot at different days to measure TRAIL-related apoptosis factors and HTNV. **(A)** HTNV S, TRAIL DR4, DR5, caspase-8, and caspase-3 mRNA expression. **(B)** HTNV NP and TRAIL-related apoptosis proteins. **(C)** DcR1/DcR2 mRNA and protein. The mRNA results shown are the average of three replicates and values represented the mean ± SD (^*^p < 0.05; ^**^p < 0.01; ^***^p < 0.001). The protein result represents one of three similar experiments. Numbers represent the relative density of the bands relative to the internal control. **(D)** Immunofluorescence images and **(E)** statistical analysis of TUNEL assay on HTNV-infected HUVECs with rTRAIL treatment. Cells were stained for virus infection [NP (sred)], for apoptosis [TUNEL (green)], and for nucleus [Hoechst (blue)]. Images data showed one of three independent experiments with similar results.

The apoptosis is an important innate antiviral response of host cells (40). To explore the role of TRAIL-mediated apoptosis in HTNV infection, we first infected HUVECs with HTNV and cultured the cells in the presence of recombinant soluble TRAIL. Our data showed that exogenous rTRAIL could reduce HTNV burden *in vitro* (Figures 3A,B). More obviously, rTRAIL could increase the expression of DR4 and DR5 (Figures 3A,B), while rTRAIL had a slight effect on the expression of DcR1 and DcR2 (Figure 3C). TRAIL also upregulated the expression of cleaved-caspase-8, total caspase-3, and cleaved-PARP (Figures 3A,B), which are involved in the apoptotic pathway. We also implemented the TUNEL staining experiment at 2 dpi (Figure 3D) and found that rTRAIL alone could not induce apoptosis in HUVEC (Figures 3D,E; compare second row to first row), while rTRAIL could induce apoptosis in HTNV-infected HUVECs (Figures 3D,E; compare fourth row to third row). Our data indicated that HTNV sensitizes HUVECs to TRAIL-mediated apoptosis, which may be closely related to anti-HTNV activity of TRAIL.

**Figure 4:**
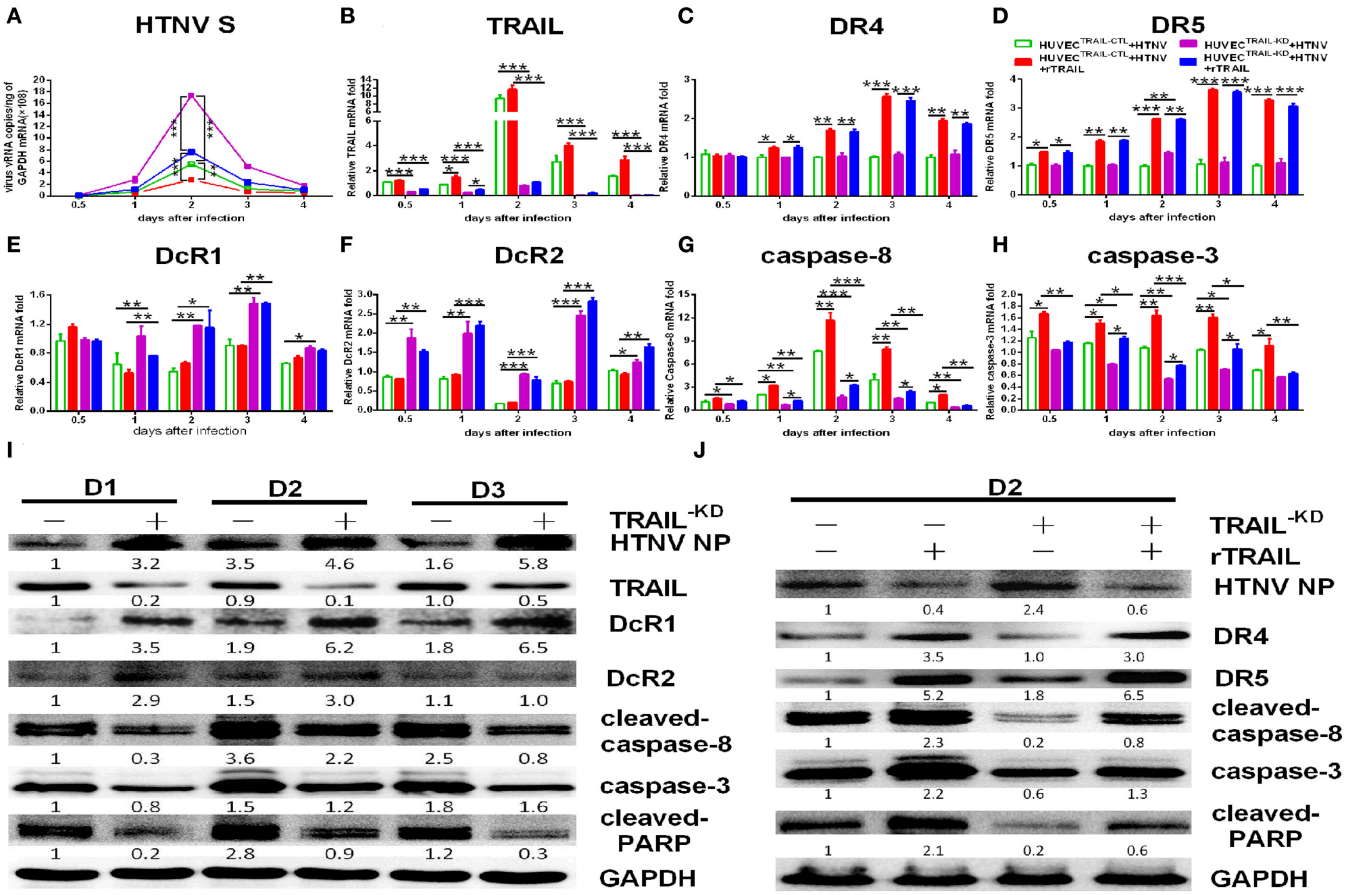
shRNA-mediated knockdown and rescue of TRAIL influence HTNV replication and apoptosis in HUVECs. HUVEC^TRAIL−KD^ cells were infected with HTNV76-118 (MOI = 1) for 2 h, treated with/without rTRAIL (40 ng/mL), and then followed by qRT-PCR and Western blot at different days to measure TRAIL-related apoptosis factors and HTNV. The mRNA results shown were the average of three replicates. Values represent the mean ± SD (^*^p < 0.05; ^**^p < 0.01; ^***^, *p* 0.001). **(A)** HTNV S mRNA, **(B)** TRAIL mRNA, **(C)** DR4 mRNA, **(D)** DR5 mRNA, **(E)** DcR1 mRNA, **(F)** DcR2 mRNA, **(G)** caspase-8 mRNA, and **(H)** caspase-3 mRNA. **(I)** HTNV NP and TRAIL-related apoptosis proteins in HTNV-infected HUVEC^TRAIL−KD^ cells at 2, 3, and 4 dpi. **(J)** HTNV NP and TRAIL-related apoptosis proteins of HUVEC^TRAIL−KD^ cells with rTRAIL rescue. Proteins were quantified using carestream software and compared with the mock group of 2 dpi; the data shown represents one of three similar independent experiments. The numbers represent the relative density of the bands relative to the corresponding control.

We further constructed lentiviruses expressing the shRNA-targeting TRAIL gene to determine its function in our study (Figure S1). The knockdown efficiency of TRAIL-specific shRNA lentivirus in HUVECs were 71.59 ± 1.86 and 87.48 ± 0.97%, as determined by qRT-PCR (Figure S1D) and Western blot (Figure S1E), respectively. As expected, silencing of TRAIL increased HTNV replication in HUVECs (Figures 4A,B,I), and the reduced expression of TRAIL significantly upregulated the expressions of DcR1 and DcR2 after HTNV infection (Figures 4E,F,I), while showing a light effect on the expressions of DR4 and DR5 (Figures 4C,D; compare white column to gray column). Further, qRT-PCR and Western blot assays show decreased TRAIL expression also resulted in lower levels of cleaved-caspase-8, total caspase-3, and cleaved-PARP (Figures 4G–I).

Furthermore, rTRAIL treatment could reverse increased viral replication induced by TRAIL knockdown in HTNV-infected HUVECs (Figures 4A,B,J). The decreased apoptotic effect shown in HTNV-infected HUVEC^TRAIL−KD^ stable cells was also reversed by exogenous rTRAIL treatment (Figures 4G,H,J), which was mainly due to the elevated expression of DR4 and DR5 (Figures 4C,D; compare black column to grid column, Figure 4J). rTRAIL could also rescue the expression of cleaved-caspase-8, total caspase-3, and cleaved-PARP at 2 dpi (Figure 4J).

Collectively, these results indicated that HTNV could trigger apoptosis through activating TRAIL-mediated pathway, and TRAIL-mediated apoptosis further suppresses viral replication.

### TRAIL-Dependent IFN-β Production Inhibits HTNV Replication

**Figure 5:**
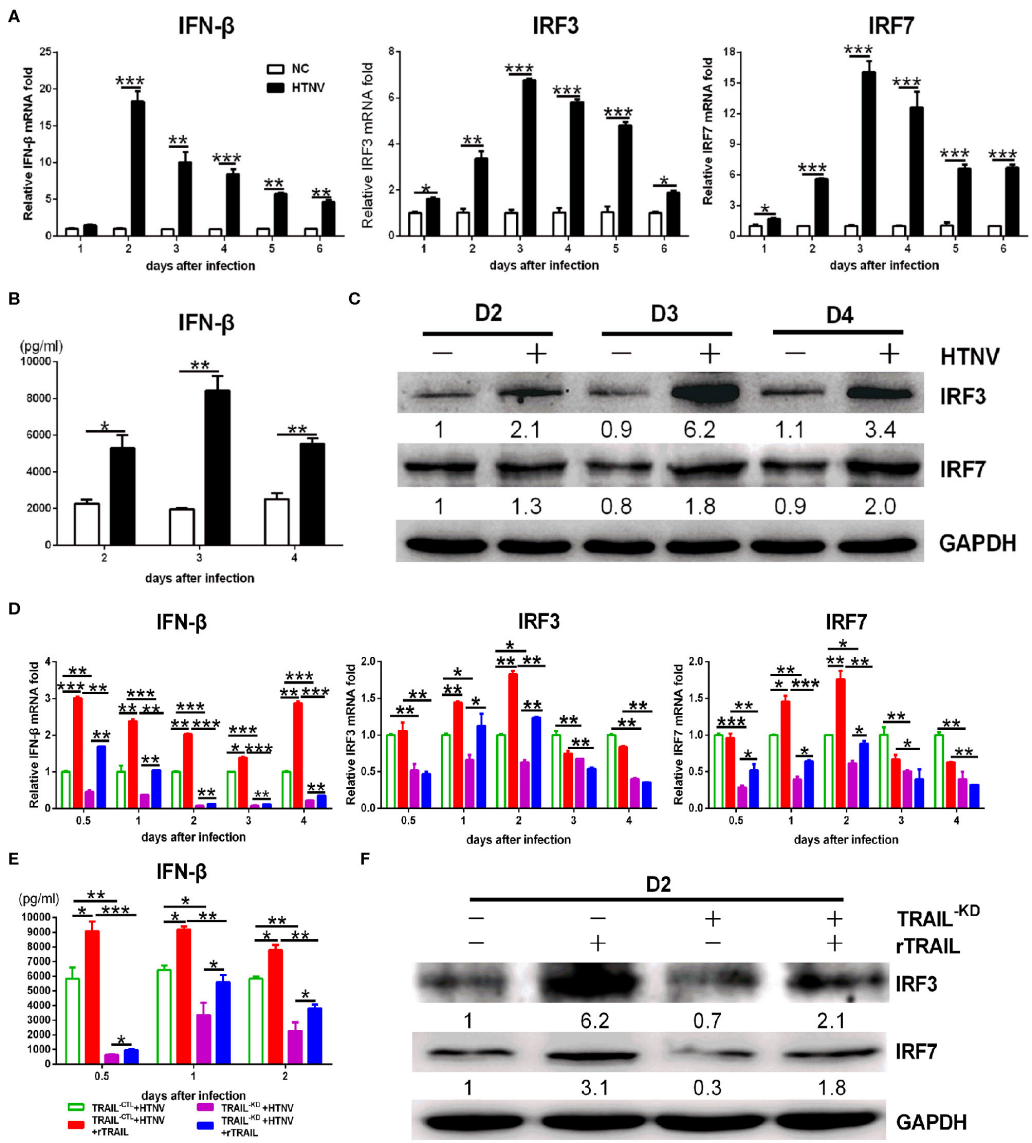
TRAIL-dependent IFN-β production suppresses HTNV replication. **(A)** IFN-β, IRF3, and IRF7 mRNA levels in HUVECs were assessed by qRT-PCR at 1, 2, 3, 4, 5, and 6 dpi. **(B)** The IFN-β in supernatant of two groups was measured by ELISA at 2, 3, and 4 dpi. **(C)** IRF3 and IRF7 proteins in cell lysate were detected by Western blot at 2, 3, and 4 dpi. **(D)** IFN-β, IRF3 and IRF7 mRNA in the rescue experiment were assessed by qRT-PCR at 0.5, 1, 2, 3, and 4 dpi. **(E)** The IFN-β in supernatant of different groups from the rescue experiment was measured by ELISA at 0.5, 1, and 2 dpi. **(F)** IRF3 and IRF7 proteins in cell lysate from the rescue experiment were detected by Western blot at 2 dpi. The results of mRNA and ELISA shown are the average of three replicates; values represent the mean ± SD (^*^p < 0.05; ^**^p < 0.01; ^***^p < 0.001). Protein data showed one of three independent experiments with similar results. Numbers represented the relative density of the bands relative to the internal control.

Next, we investigated whether TRAIL could exert additional anti-HTNV activity distinct from apoptosis. Viral infection triggers the production of a vast amount of antiviral IFNs. We observed that IFN-β, rather than IFN-α, could be easily detected by qRT-PCR in HTNV-infected HUVECs (around 16-fold at 2 dpi; Figure 5A). The secreted IFN-β in the supernatant of HTNV-infected HUVECs was also significantly upregulated at 2–4 dpi (Figure 5B). The time-course detection showed that HTNV obviously promoted the mRNA levels and proteins expression of IRF3 and IRF7 (Figures 5A,C). These data indicate that HTNV infection induced type I IFNs and transcription factors in HUVECs. Based on the HTNV-infected HUVEC^TRAIL−KD^ stable cells, we found that silencing of TRAIL significantly decreased HTNV-induced IFN-β production (Figures 5D,E; compare white column to gray column), as well as the expression of its transcription factors (IRF3 and IRF7) during HTNV infection (Figures 5D,F; compare white column to gray column). Exogenous rTRAIL could partially restore the expression of IRF3, IRF7, and IFN-β at early stage of viral infection (0.5–2 dpi, respectively) (Figures 5D–F; compare gray column to grid column). The silencing of TRAIL could also decrease the protein level of IRF3 and IRF7, while exogenous rTRAIL enhanced the protein expressions of IRF3 and IRF7 (Figure 5F). These data imply the direct role of TRAIL in HTNV-induced IFN-β expression, suggesting that TRAIL may activate multiple antiviral pathways.

### Protective Efficacy of TRAIL in Suckling Mice

**Figure 6:**
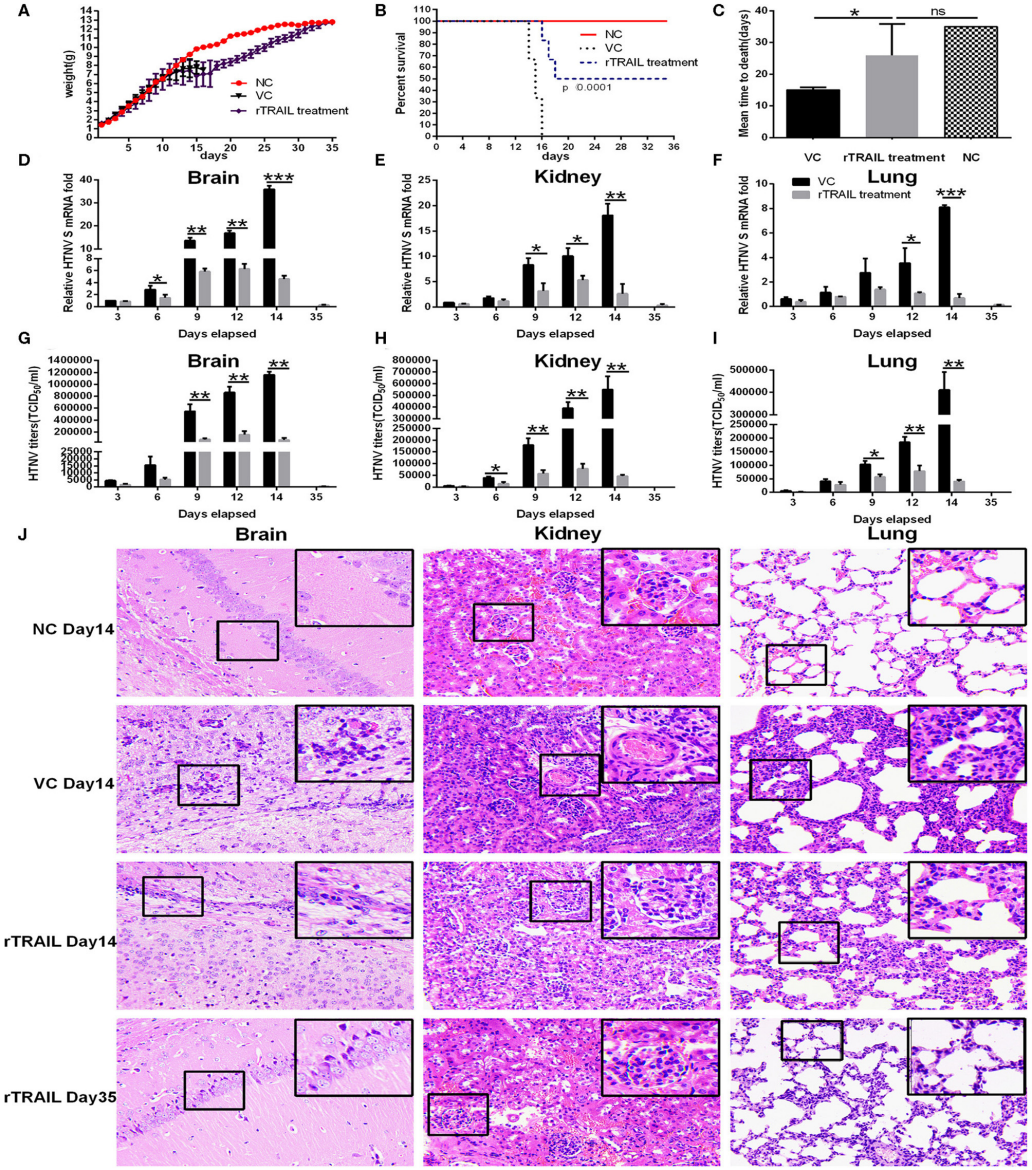
Effect of rTRAIL on lethal HTNV challenge model. Newborn BALB/c mice were infected with 50 × LD_50_ of HTNV76-118 followed with or without intraperitoneal injection of rTRAIL (0.5 μg/g/day) for 7 days. **(A)** The effects of rTRAIL on HTNV infection in mice were determined by body weight change from 0 to 35 dpi (*n* = 12 in each group). Mice were weighted individually every day. **(B)** The effects of rTRAIL on HTNV virulence in mice were determined by survival rate (*n* = 12 in each group). **(C)** Effect of rTRAIL on MTD of mice infected with HTNV. **(D–F)** Mice were sacrificed at 3, 6, 9, 12, 14, and 35 dpi, respectively, and brains, kidneys, and lungs were collected for qRT-PCR to assess HTNV S segment levels. The experiment was carried out three times and values represent the mean ± SD (^*^p < 0.05; ^**^p < 0.01; ^***^p < 0.001). **(G–I)** Mice were sacrificed at 3, 6, 9, 12, 14, and 35 dpi, respectively, and brains, kidneys, and lungs were collected to assess HTNV titers by Reed-Munch method. **(J)** H & E staining for mouse brain, kidney or lung specimens was performed.

To assess the contribution of TRAIL in antiviral response, we examined the *in vivo* effect of TRAIL against HTNV infection. In this experiment, the newborn BALB/c mice were infected with HTNV followed with or without intraperitoneal injection of rTRAIL. Compared to the viral control group, rTRAIL reduced clinical signs, which included ruffled fur, tendencies to huddle, paralysis of hind leg and spasm (data not shown). All the mice in viral control group died with an MTD (mean time to death) of 15 ± 0.3651 days, while rTRAIL increased survival rate and prolonged mouse survival time (Figures 6B,C). It is worth noting that the weight change of the mice between the virus group and the rTRAIL treatment group showed no significant difference before 16 dpi. Due to the protective effect of rTRAIL, half of the mice in the rTRAIL-treated group survived and the body weight gradually recovered to normal at 35 dpi (Figure 6A).

Next, we analyzed the effect of TRAIL on viral replication in different tissues. Notably, rTRAIL decreased viral replication in the brains, kidneys, and lungs about 5- to 10-fold. The most significant inhibitory effect appeared at 14 dpi, with 87.14 ± 3.29, 85.58 ± 1.17, and 91.48 ± 3.35% in brains, kidneys and lungs, respectively (Figures 6D–F). As expected, rTRAIL treated mice showed considerably lower HTNV titers in the brains, kidneys, and lungs at 3, 6, 9, 12, and 14 dpi (Figures 6G–I). In addition, we observed that HTNV infection caused anabatic tissue injury (inflammatory cell infiltration, alveolar enlargement) in brains, kidneys, and lungs, and rTRAIL treatment could reduce the tissue injury caused by HTNV at the late stage of infection (Figure 6J).

**Figure 7:**
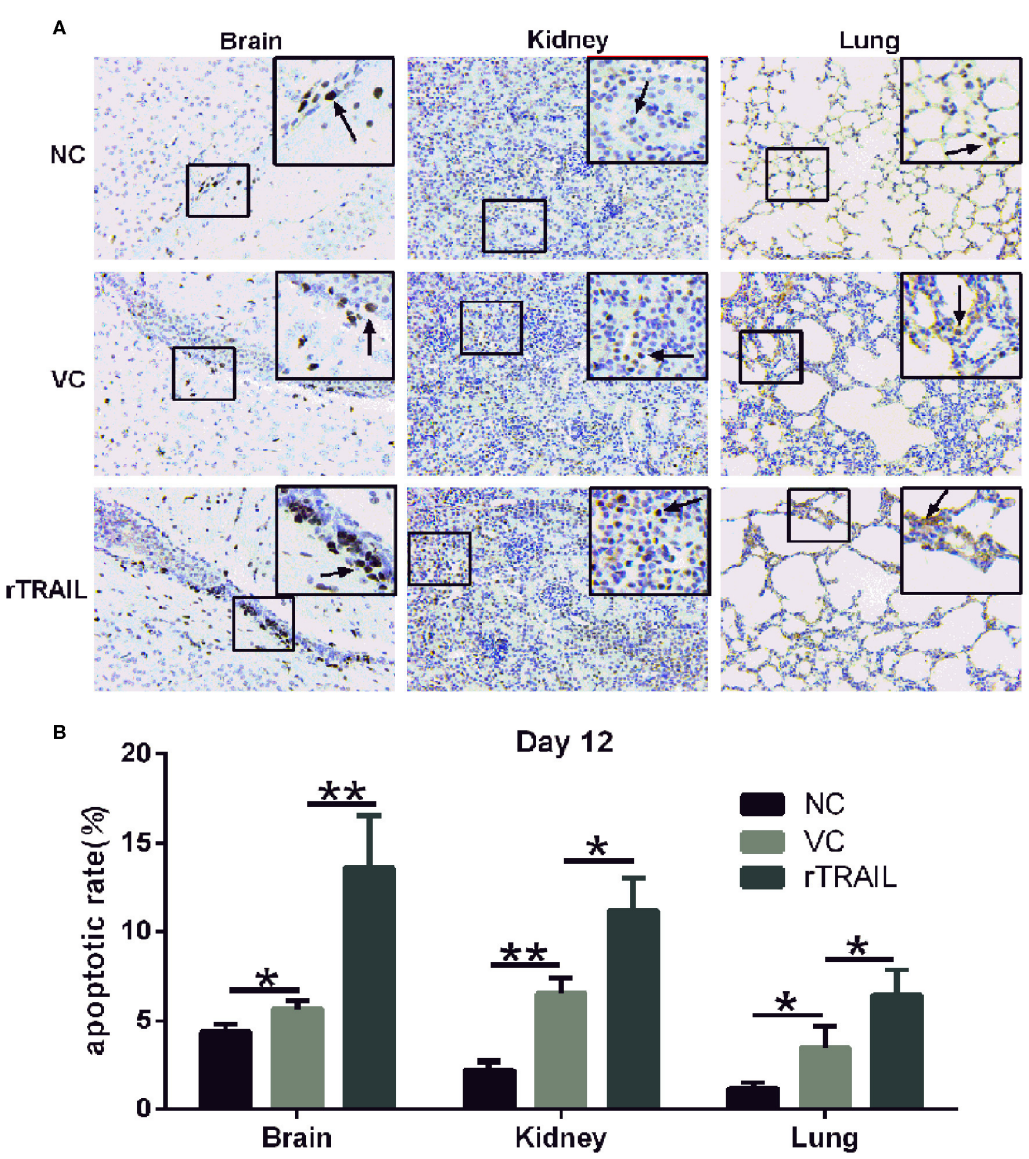
rTRAIL increases tissue apoptosis in HTNV-infected mice. **(A)** Representative TUNEL staining images of for mouse brain, kidney or lung specimens. The mice were sacrificed at 12 dpi and prepared for TUNEL assay to detect the tissue apoptosis. Black arrow notes apoptotic cells. **(B)** Analysis of TUNEL apoptosis pictures. Each slide was randomly selected for eight 200-fold visuals and TUNEL apoptotic images were analyzed by Image pro-plus 6.0 software. The percentage of TUNEL-positive cells was calculated and analyzed (^*^p < 0.05; ^**^p < 0.01). Data shown represent one of three similar independent experiments.

As TRAIL was closely associated with apoptosis, we performed the TUNEL assay to identify cells in the late apoptotic stage (Figure 7A), which exhibited extensive DNA degradation. Data showed there was an increase in the density of TUNEL-positive cells in the brains (29.07 ± 8.06%), kidneys (192.73 ± 61.55%), and lungs (194.7 ± 25.94%) in mice infected by HTNV compared with normal controls (Figure 7B), which demonstrated HTNV-induced apoptosis *in vivo*. Moreover, TUNEL-positive cells in brains, kidneys, and lungs from rTRAIL treated mice were significantly increased, compared to virus controls, by 141.10 ± 30.68, 85.72 ± 30.85, and 71.55 ± 18.11%, respectively (Figure 7B). The above findings indicated that TRAIL can suppress HTNV replication *in vivo* through inducing the apoptosis of cells.

## Discussion

TRAIL, an immune surveillance factor, has shown promising successes in killing cancer cells through specifically inducing apoptosis of cancer cells and no effects on normal cells (41). Unlike its tumor-suppressive function, TRAIL plays miscellaneous roles in virus infection, protective or pathogenic, antiviral or proviral, depending on the virus and tissue-specific factors. Here we found a new function for TRAIL associated with host defense against hantavirus.

Clinical evidence has shown that HTNV infection is related to the upregulation of sTRAIL in HFRS patients (34, 42). We document that HTNV infection leads to an increase of TRAIL expression in HUVECs, and TRAIL exhibits striking antiviral activities by inducing apoptosis *in vitro*. To further explore the mechanism of HTNV-triggered TRAIL-dependent apoptosis, we confirm that HTNV also induces TRAIL-dependent apoptosis in HUVECs by downregulating the decoy receptor expressions, which can't induce apoptosis once TRAIL is bound, because of a lacking death domain in the intracellular region (43). The luciferase reporter assay further proved that the S or M segments of HTNV could downregulate the transcription of DcR1 and DcR2. Our results were consist with data reported for HIV Env-pseudotyped virus (44). Interestingly, the previous Affymetrix DNA Arrays analysis also showed the increasing expression of TRAIL and reduction level of DcR2 in HTNV-infected HUVECs (45). Wang et al. demonstrated that DcR1 and DcR2 overexpression blocks TRAIL-mediated apoptosis via their competitive TRAIL binding with DR4 and DR5 (46). By the same token, reduction of DcR1 and DcR2 in HTNV-infected HUVECs results in overflowing TRAIL binding with DR4 and DR5, thereby promoting cell apoptosis. Considering the TRAIL-resistance of HUVECs, we concluded that the dynamic expression of TRAIL and its receptors after HTNV infection, as well as the relative abundance of each distinct receptor under the TRAIL treatment, eminently contributed to modulating sensitivity to TRAIL-mediated apoptosis in the primary HUVECs.

Consistent with our results, CMV (47), HCV (37), and HIV (44) have been demonstrated to trigger TRAIL-mediated apoptosis. We also showed that exogenous TRAIL can directly increase the expressions of DR4/DR5 and then further enhance their downstream caspase-8-dependent apoptotic pathway in HTNV-infected HUVECs, which is a conventional mode of action present in various virus infection, such as reovirus (48) or RSV (24). However, the latest research (42) has reported that HTNV protects infected cells from TRAIL-mediated NK killing by downregulating DR5. In contrast, we reported that HTNV-infected HUVECs exhibited slight change of pro-apoptotic receptors (DR4 and DR5) and significant decline of anti-apoptotic receptors (DcR1 and DcR2). Our results are also in agreement with previous studies showing that HBV (22, 49), HCV (37), and HIV (44) infection could trigger TRAIL-mediated apoptosis by upregulating DR4/DR5 or downregulating DcR1/DcR2. Both Sola-Riera et al. and the present study showed TRAIL upregulation on HUVECs. However, Sola-Riera et al. demonstrated resistance of HTNV-infected A549 cells to TRAIL-induced apoptosis, whereas our data indicate that HTNV-infected HUVECs undergo apoptosis after treatment with rTRAIL (Figures 3A,B,D,E; compare fourth row to third row). We assume that the different cell types used (HUVECs, primary cells, in the case of the present study vs. A549 cells, a transformed cell line, in the case of Sola-Riera et al.) could explain the difference. Another concern is the source or genetic background of the primary HUVECs, which could largely affect the experimental results from different research. For example, Sola-Riera et al. demonstrated that the HTNV does not induce autophagy in HUVECs (obtained from Lonza). However, the latest research (50) published in Cell Reports also revealed that HTNV could dynamically regulate the host autophagy process for viral benefits in HUVECs (obtained from ScienCell). Therefore, factors, such as the source or genetic background of the primary HUVECs, the use of a transformed cell line (A549 cells), and the use of mouse-adapted or non-adapted HTNV with an uncertain passage history, need to be taken into account when comparing results from different laboratories. Nevertheless, combined with the results obtained in the animal experiment, our results indicate that HTNV infection induces TRAIL expression and its related apoptosis in tissue culture cells and in a mouse model. More importantly, TRAIL mediated a novel anti-HTNV activity through apoptosis *in vitro* and *in vivo*.

It has been reported that the viral structural or non-structural proteins play important roles in regulating the expression of TRAIL and its receptors, such as the HBV X protein (22), HIV-1 Tat protein (51), and adenovirus E3 protein (52). In the meantime, emerging studies suggested hantavirus proteins also can interact with host cell proteins, in particular N protein. PUUV N protein can interact with the Daxx protein, a Fas-mediated apoptosis enhancer (11), which also functions as transcription repressor. Kaukinen et al. hypothesized that the binding of hantaviral N proteins with Daxx in the cytoplasm could restrain Daxx-mediated transcriptional repression and trigger the Fas–apoptosis pathway in hantavirus-infected cells (53). Researchers further revealed that hantavirus infection may disturb the DAXX-PML colocalization in nuclear bodies, which disrupt their cooperation to mediate apoptosis in ANDV and HTNV infected HUVECs (12). Tula (54) and HTNV (55) N proteins are able to interact with small ubiquitin-related modifier-1 (SUMO-1), which can modulate multiple cellular processes including cell growth and signal transduction. We observed in this study that NP and GP proteins of HTNV were capable of promoting the transcription of TRAIL and its pro-apoptotic receptors, which linked HTNV structural proteins with TRAIL-mediated cells apoptosis. However, more research is needed to determine the real relationships of NP and GP with apoptosis. Taken together, we demonstrated that HTNV induces TRAIL-mediated apoptosis in endothelial cells. During viral infection, apoptosis can decrease the injurious viral load and hinder the spread of progeny virus (56). Thus, TRAIL-mediated apoptosis implies TRAIL is also a kind of antiviral proteins against HTNV infection.

Besides apoptosis induction, we further confirmed the role of TRAIL on IFN expression induced by HTNV infection. We also demonstrated that the IFN-β production is associated with TRAIL-dependent upregulation of transcription factors (IRF3 and IRF7), which are able to activate IFN production (57). It is well-known that after binding TRAIL, the intracellular region of pro-apoptotic DR4/DR5 recruits the intercellular adaptor proteins, such as the Fas-associated death domain (FADD), TNF receptor type 1-associated death domain protein (TRADD), or receptor-interacting protein-1 (RIP1), to trigger multiple cell signaling (58). Balachandran et al. demonstrated that FADD suppresses viral replication through TBK-1-mediated IRF3 activation, which implies an alternative FADD-dependent IFN production in innate immune pathway besides the Toll pathway (59). Previous studies have found that the dimerization of MAVS, a mitochondria-associated adaptor protein, can recruit TRADD to induce the phosphorylation of IRF3 and IRF7 and their downstream events, type I IFN production (60). It is worth noting that TRADD and FADD are not only involved in the TRAIL-induced apoptosis (61, 62), but also mediate multiple signaling events, especially IFN production. IFN responses provide the initial defense against viral invasions in the innate immune system. Based on our results, we assume that TRAIL/IFN signaling may involve in FADD or TRADD recruitment and then trigger subsequent IRF3/IRF7 activation, which offers another explanation for anti-HTNV effect of TRAIL except for apoptosis.

It is important to consider whether TRAIL exerts anti-HTNV activities *in vivo*. Here, we used the lethal HTNV-infected suckling mice model previously established by our laboratory (36). The results showed that TRAIL exhibited a significant protective effect on HTNV-infected suckling mice through significantly decreasing tissue virus load and alleviating pathologic lesions. Our results are consistent with previous reports that administration of anti-TRAIL antibody can significantly elevate viral titer in the heart of ECMV-infected mice (32) or delay virus clearance in the lung of influenza virus infected mice (63). Brincks et al. employed an influenza virus infection model of TRAIL^−/−^ mice, and demonstrated that TRAIL deficiency increases morbidity and virus titers, which are associated with the decreased CTL response (64). In our case, TRAIL could enhance HTNV-induced apoptosis in the brains, kidneys, and lungs, which highlights the importance of TRAIL-mediated apoptosis in anti-HTNV activity. Further studies will be needed to elucidate which signaling molecules are involved in these events.

TRAIL has drawn attention for its potential crucial role in the defense against HTNV infection. We have shown that HTNV induces TRAIL-dependent extrinsic apoptosis in HUVECs involving downregulation of the decoy receptors. Furthermore, TRAIL-dependent IFN production is promoted by IRF3/IRF7 activation and ultimately suppresses viral replication via its downstream innate immune response. The protection provided by TRAIL in mice suggested that TRAIL may be a novel target for curing severe hantavirus infection.

## Data Availability Statement

All datasets generated for this study are included in the article/Supplementary Material.

## Ethics Statement

The animal study was reviewed and approved by Institutional Animal Care and Use Committee (Wuhan, China).

## Author Contributions

Q-ZC, H-RX, and WH conceived and designed the experiments. H-RX, FL, NZ, and WH contributed to the funding acquisition. Q-ZC, XW, FL, NL, NZ, SL, YZ, C-JZ, and M-RW performed the experiments. Q-ZC, XW, and FL analyzed the data. Q-ZC wrote original draft. H-RX, H-TH, Y-ZZ, and WH revised the paper. All authors approved the final version of the manuscript.

## Conflict of Interest

The authors declare that the research was conducted in the absence of any commercial or financial relationships that could be construed as a potential conflict of interest.
