# Supplementary material for: HTNV Sensitizes Host Toward TRAIL-Mediated Apoptosis—A Pivotal Anti-hantaviral Role of TRAIL
Source: Front Immunol. 2020 Jun 19;11:1072. doi: 10.3389/fimmu.2020.01072 (PMC7317014; doi:10.3389/fimmu.2020.01072)
Supplement: Supplementary file 1 [file Data_Sheet_1.DOCX]

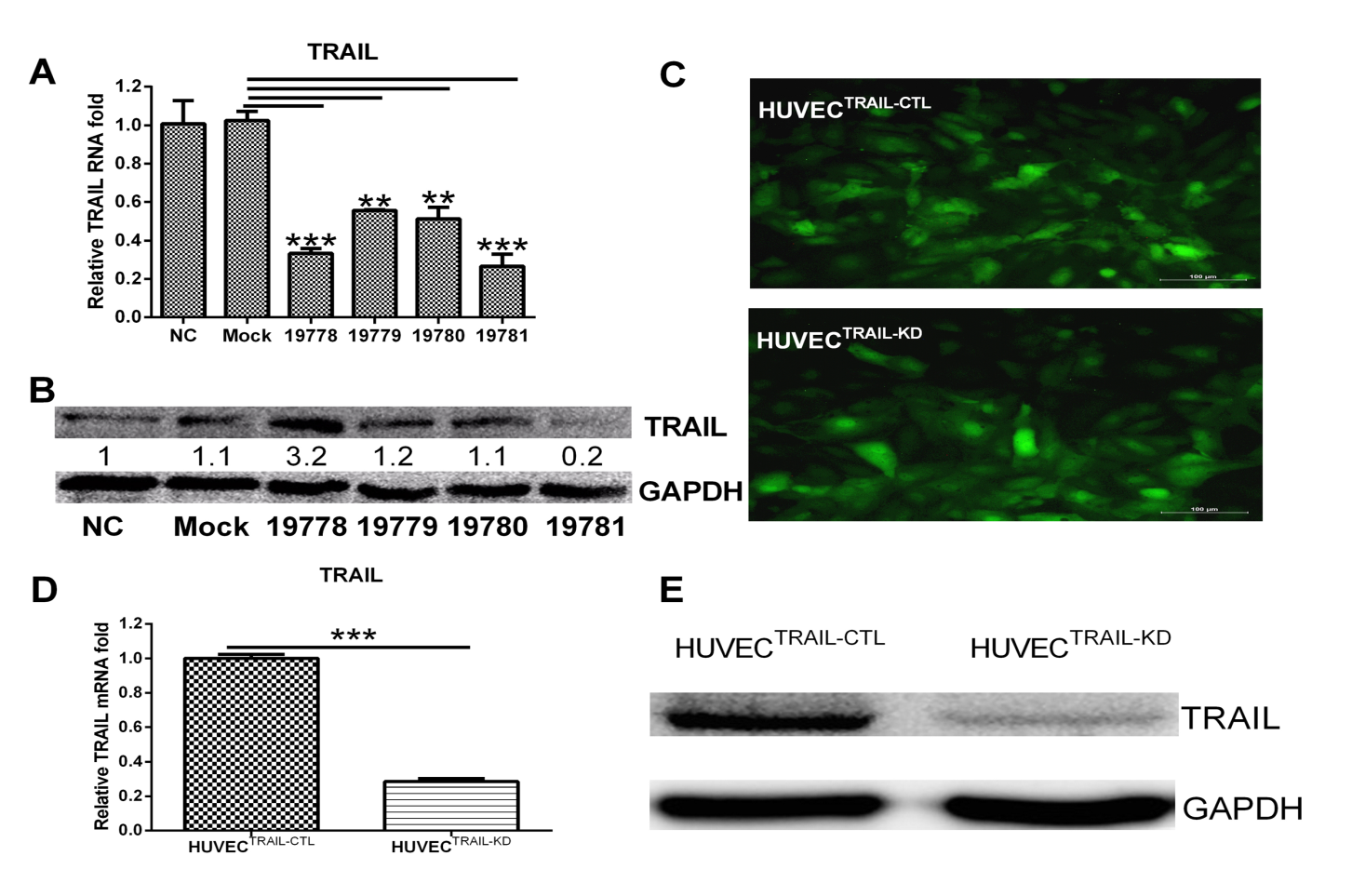


**Fig S1. Establish stable HUVECs strain interference of TRAIL gene and protein.** Four shRNA (shRNA 19778, 5′-AACAAATGAGCACTTGATA-3′; shRNA 19779: 5′-ACAAACAAATGGTCCAATA-3′; shRNA 19780: 5′-ATTTCTACAGTTCAAGAAA-3′; and shRNA 19781: 5′-TGTAACTTACGTGTACTTT-3) targeting human TNFSF10 gene (Gene Bank accession NM_003810) were tested for their abilities to silence TRAIL in 293T cells. (A) TRAIL mRNA, (B) TRAIL protein. HUVECs were transduced with lentiviral particles at MOI of 1. The transduction efficiency was observed and recorded under a fluorescence microscope (Nikon TE2000) after 48 h post infection (C). The interference efficiency was determined by qRT-PCR (D) and Western blot (E). The experiment was carried out three times and protein data were from one of three similar independent experiments. Values represented the mean ± SD (**p* < 0.05, ***p* < 0.01).

**

Fig S2. The uninfected HUVECs were simultaneously collected as blank control for Flow cytometry analysis**. (A) The scatter plot represented the selected analyzing cell population. (B) & (C) Histogram of the selected analyzing cell population. The experiment was repeated three times and data represented one of three separate experiments.

**

**

**Fig S3: Level of HTNV infected endothelial cells.** (A) HTNV-infected HUVECs at 3 dpi were fixed and stained green for HTNV nucleocapsid protein with the mAb ab20309, and Hoechst for nuclear staining. Images data showed one of three independent experiments. (B) Percentage of infected cells were then quantified using fluorescence microscopy. Eight horizons were selected randomly to evaluate HTNV-infected rate. Data shown represented mean ± SEM from 3 independent experiments.
